# Improvement of Enzymatic Glucose Conversion from Chestnut Shells through Optimization of KOH Pretreatment

**DOI:** 10.3390/ijerph18073772

**Published:** 2021-04-04

**Authors:** Kang Hyun Lee, Soo Kweon Lee, Jeongho Lee, Seunghee Kim, Chulhwan Park, Seung Wook Kim, Hah Young Yoo

**Affiliations:** 1 Department of Biotechnology, Sangmyung University, 20, Hongjimun, 2-Gil, Jongno-Gu, Seoul 03016, Korea; oys7158@naver.com (K.H.L.); jeongholee0601@gmail.com (J.L.); kimseunghee02@naver.com (S.K.); 2 Department of Chemical and Biological Engineering, Korea University, 145 Anam-Ro, Seongbuk-Gu, Seoul 02841, Korea; sookweon@korea.ac.kr; 3 Department of Chemical Engineering, Kwangwoon University, 20 Kwangwoon-ro, Nowon-gu, Seoul 01897, Korea

**Keywords:** chestnut shells, statistical optimization, KOH pretreatment, enzymatic hydrolysis, food processing wastes

## Abstract

Worldwide, about one-third of food produced for human consumption is wasted, which includes byproducts from food processing, with a significant portion of the waste still being landfilled. The aim of this study is to convert chestnut shells (CNSs) from food processing into a valuable resource through bioprocesses. Currently, one of the highest barriers to bioprocess commercialization is low conversion of sugar from biomass, and KOH pretreatment was suggested to improve enzymatic digestibility (ED) of CNS. KOH concentration of 3% (*w*/*w*) was determined as a suitable pretreatment solution by a fundamental experiment. The reaction factors including temperature, time and solid/liquid (S/L) ratio were optimized (77.1 g/L CNS loading at 75 °C for 2.8 h) by response surface methodology (RSM). In the statistical model, temperature and time showed a relatively significant effect on the glucan content (GC) and ED, but S/L ratio was not. GC and ED of the untreated CNS were 45.1% and 12.7%, respectively. On the other hand, GC and ED of pretreated CNS were 83.2% and 48.4%, respectively, and which were significantly improved by about 1.8-fold and 3.8-fold compared to the control group. The improved ED through the optimization is expected to contribute to increasing the value of byproducts generated in food processing.

## 1. Introduction

According to the Food and Agriculture Organization of the United Nations (FAO), a huge amount of food is produced every year for human consumption, however, about one-third is lost or wasted. Wastes generated from food processing also have a significant proportion, but most of them are landfilled. Since landfill or incineration is not desirable due to environmental pollution, the development of recycling technologies is urgently required [1]. Although the concept of biorefining has emerged to produce value-added substances including fuels, chemicals and substances from biomass, economical biorefinery design requires the supply of inexpensive raw materials such as agri-food waste [2,3].

Chestnut has received attention in the European food industry because it is a resource to produce marron glace and chestnut flour used for gluten-free diets [4]. According to a report by Pinto et al., world chestnut production was estimated to be 2.4 million tons in 2019 [5]. During the chestnut processing, outer shells (8.9–13.5%) and inner shells (6.3–10.1%) are generated as the residues [6]. These residues are commonly used for animal feed, compost, cosmetic ingredient and adhesive [7,8]. However, most of the chestnut shells (CNSs) have not been utilized and are disposed of as waste. It is necessary to suggest a new utilization plan for CNS. CNSs have been reported to consist of 21.5–29.0% cellulose, 16.2–26.0% hemicellulose and 35.2–35.4% lignin [9,10]. Due to these constituents of CNS, it was expected to be suitable as a raw material for biorefinery.

Several processes including pretreatment, saccharification and fermentation should be performed to utilize lignocellulosic biomass in biorefinery [11]. It has been reported that pretreatment and enzymatic hydrolysis account for 35% of the total biorefinery cost [12]. In order to reduce the overall process cost, it is significant to derive the optimum reaction conditions for each step. Pretreatment of biomass is a key process required to improve the recovery of fermentable sugars in enzymatic hydrolysis [13]. Various pretreatment (physical, chemical and biological) of lignocellulosic biomass have been conducted to remove the lignin, which inhibits the enzymatic hydrolysis, and to enhance the depolymerization and dissociation of fermentable sugar [14]. Chemical pretreatment has been widely used because of its advantages of low process cost and high sugar recovery [15]. Alkali pretreatment is carried out under mild conditions with less corrosive reagents including potassium hydroxide (KOH), sodium hydroxide (NaOH) and calcium hydroxide (Ca(OH)_2_), and generates less fermentation inhibitory compounds compared to acid pretreatment [16]. The mechanism of alkali pretreatment is that saponification of intermolecular ester bonds by alkali reagents breaks the crosslinks between lignin, cellulose and hemicellulose, causing delignification [17]. The delignified biomass increases biodegradability and enzymatic accessibility, which leads to improved enzymatic hydrolysis efficiency [18]. Fermentable sugars produced by enzymatic hydrolysis are suitable for microbial fermentation because they have no fermentation inhibitory compounds such as furfural and 5-hydroxymethyl furfural (HMF) [19]. Various studies have been reported that have successfully produced the value-added materials such as bioethanol, cordycepin and carotenoid using these fermentable sugars for microbial fermentation [18,19,20,21].

In this study, to improve the enzymatic digestibility of CNS, the conditions of KOH pretreatment were optimized by the statistical method. Before optimization, fundamental effect of KOH concentration on chemical composition of CNS and the efficacy of enzymatic hydrolysis were investigated, and the desirable concentration of KOH was determined. In addition, the pretreatment conditions of reaction time, temperature and S/L ratio were optimized by response surface methodology (RSM). Under optimum conditions, the pretreated CNS was enzymatically hydrolyzed to produce glucose.

## 2. Materials and Methods

### 2.1. Materials

CNSs were purchased from Cheongmyeongyagcho (Chungju-si, Chungcheongbuk-do, Korea). CNSs were dried at 105 °C for 48 h to remove moisture. The dried CNSs were pulverized to a size of 425 μm and stored in plastic bags at 4 °C. Celluclast^®^ 1.5 L (cellulase) and Cellic^®^ CTeC2 (cellobiase) were purchased from Sigma-Aldrich (St. Louis, MO, USA). Potassium hydroxide (KOH), sulfuric acid (H_2_SO_4_), calcium carbonate (CaCO_3_) and citric acid monohydrate were purchased from Duksan Chemical (Ansan-si, Gyeonggi-do, Korea).

### 2.2. KOH Pretreatment of Chestnut Shells

In order to investigate the KOH concentration suitable for CNS pretreatment, KOH solutions were prepared in various concentrations (0%, 1%, 2%, 3%, 4% and 5%, *w*/*w*). A 0% KOH concentration means that CNSs were pretreated using deionized water (DW). KOH pretreatment was performed with 100 g/L solid loading at 70 °C for 2 h. After pretreatment, the solid fractions were neutralized with DW until pH 7 and dried at 105 °C for 48 h. All experiments were carried out in triplicate to obtain the standard deviations. Solid recovery (SR) after pretreatment was calculated by the following equation:

Solid recovery, SR (%) = (weight of dried CNS after KOH pretreatment/weight of initial dried CNS) × 100(1)

### 2.3. Enzymatic Hydrolysis of Chestnut Shells

CNSs were hydrolyzed with 60 filter paper units (FPUs) cellulase/g-biomass and 30 cellobiase units (CBUs) cellobiase/g-biomass at 50 °C for 72 h in a 50 mM sodium citrate buffer (pH 4.8) [22]. One FPU and CBU were defined as the amount of enzyme that released 1 μmol glucose per min under the standard assay conditions. All experiments were carried out in triplicate to obtain the standard deviations.

### 2.4. Experimental Design and Statistical Optimization

In order to optimize the pretreatment conditions for CNS, the central composite design (CCD) of RSM was carried out using Design-Expert 7 software (Stat-Ease Inc., Minneapolis, MN, USA). The CCD shows the interaction between the independent factors affecting the response factor using statistical and mathematical models. Table 1 shows three independent factors (temperature, time and S/L ratio) divided into five levels (−2, −1, 0, 1 and 2) for the CCD. The range of factors was as follows: temperature (*X*_1_), 0–100 °C; time (*X*_2_), 0–4 h and S/L ratio (*X*_3_), 50–150 g/L. All experiments were carried out in triplicate to obtain the standard deviations.

The experimental results were analyzed by analysis of variance (ANOVA). Each factor and their interactions were determined by applying the following equation:

*Y* = *β*_0_ + ∑ *β*_i_*X*_i_ + ∑ *β*_ij_*X*_i_*X*_j_ +∑ *β*_ii_*X*_i_^2^ (2)

where *Y* is the response factor, *X_i_* and *X_j_* are the independent factors, *β*_0_ is the offset term, *β_i_* is the first order model coefficient, *β_ii_* is the quadratic coefficient for the factor *i* and *β_ij_* is the linear model coefficient for the interaction between factors *i* and *j*, respectively [23].

### 2.5. Analytical Methods

The chemical compositions of CNS were determined by referring to the National Renewable Energy Laboratory (NREL) analytical procedure (TP-510-42618) [24]. In 3 mL of 72% (*w*/*w*) H_2_SO_4_, 0.3 g CNS were immersed at 30 °C for 2 h. DW was added to each sample to dilute the acid concentration to 4%, and then reacted in an autoclave (VS-1221, Vision Scientific, Daejeon, Korea) at 121 °C for 1 h. After the reaction, each sample was neutralized using CaCO_3_ and the supernatant was filtered using 0.22 μm syringe filter for high liquid performance chromatography (HPLC) analysis. The HPLC analysis conditions to investigate the chemical compositions of CNS and the concentration of glucose released by enzymatic hydrolysis were as follows: Shodex SUGAR SH1011 H^+^ ion exclusion column (300 mm × 8 mm, Shodex, Japan); refractive index detector (RID-10A, Shimadzu, Japan); mobile phase of 0.005 N H_2_SO_4_; flow rate of 0.6 mL/min; temperature of column at 50 °C and injection volume of 20 μL. Glucan content (GC) and enzymatic digestibility (ED) of samples were calculated by the following equation:

Glucan content, GC (%) = (weight of glucan in dried CNS/weight of dried CNS) × 100(3)

Enzymatic digestibility, ED (%) = (weight of glucose released/(weight of glucan × 1.1)) × 100(4)

where 1.1. is the conversion factor of glucan to glucose.

## 3. Results and Discussion

### 3.1. Effect of KOH Concentration on Pretreatment of Chestnut Shells

CNS is composed of 63.7% carbohydrate and 9.4% protein, which can be utilized as carbon and nitrogen sources in fermentation. The carbohydrate content includes 45.1–57.8% glucan and 2.4–5.9% XMGA. XMGA means hemicellulose composed of xylan, mannan, galactan and arabinan. Additionally, 1.2% lipid and 2.4% ash were present in CNS (Table 2). Worldwide, about 2.4 million tons of chestnuts are produced annually [5], and the production of CNS is estimated to be about 365–566 thousand tons per year. Therefore, the potential of CNS that can be utilized as a carbon source in biorefinery was estimated to be about 173–361 thousand tons.

**Figure 1:**
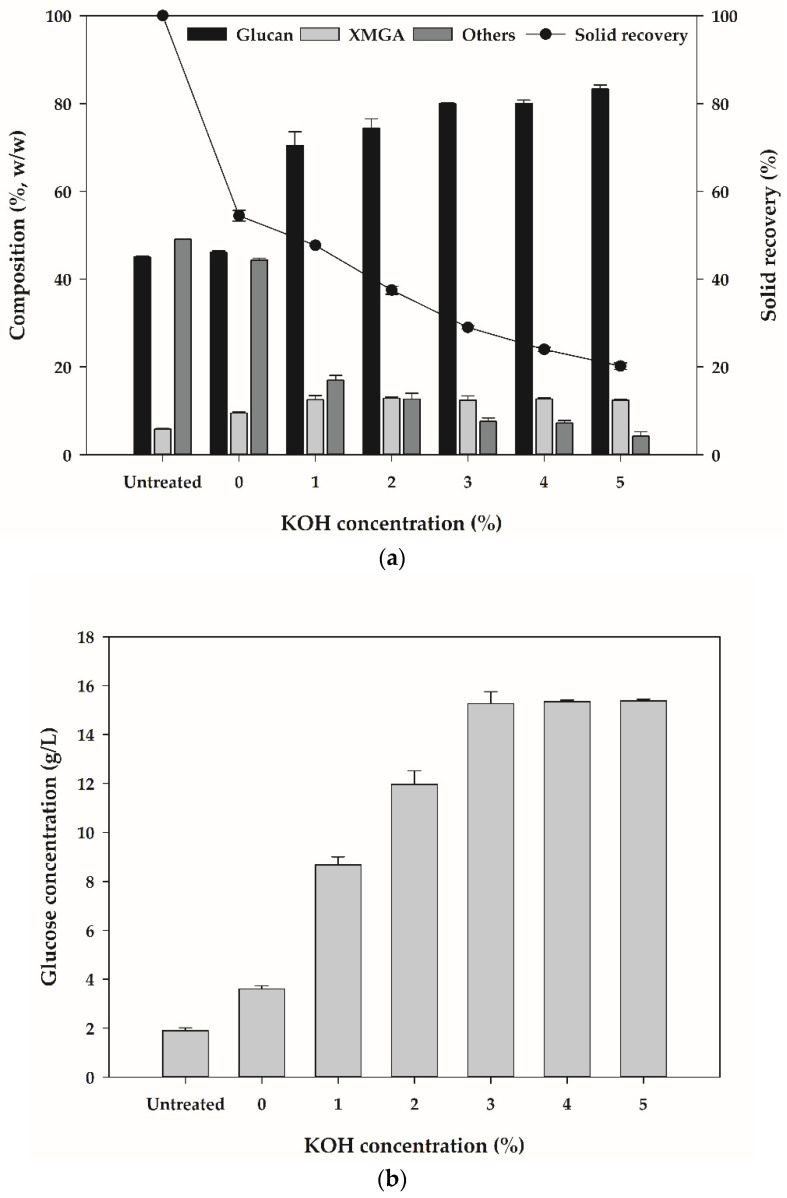
Effect of KOH pretreatment of chestnut shells (CNSs) on chemical composition (**a**) and the released glucose concentrations after the enzymatic hydrolysis (**b**).

Figure 1a shows the chemical compositions and solid recovery after KOH pretreatment of CNS. Untreated CNS consist of 45.1% glucan, 5.9% XMGA and 49.1% others. After the pretreatment using DW (0% KOH concentration) at 70 °C for 2 h, the chemical compositions of CNS were as follows: 46.1% glucan, 9.5% XMGA and 44.4% others. Pretreatment using DW did not significantly affect the complex structure of CNS and SR decreased to 54.5%. Obeng et al. reported that low SR disrupts the sugar recovery from biomass during enzymatic hydrolysis [25]. These results indicate that pretreatment using DW was not suitable for CNS pretreatment. GC following KOH pretreatment was found to be 70.4%, 74.4%, 80.1%, 80.2% and 83.3% at KOH concentrations of 1%, 2%, 3%, 4% and 5%, respectively. SR was found to be 47.7%, 47.5%, 29.0%, 24.0% and 20.2% at KOH concentration of 1%, 2%, 3%, 4% and 5%, respectively, and decreased steadily as the KOH concentration increased. KOH pretreatment significantly increased GC, and in particular, GC reached above 80.0% with a KOH concentration of 3% or more. These results are consistent with previous studies reported by Jiang et al. and Yan et al. that GC increases and SR decreases with increasing alkali concentration in biomass pretreatment [26,27].

Figure 1b shows the released glucose concentrations after enzymatic hydrolysis of pretreated CNS. The glucose concentrations released by enzymatic hydrolysis of untreated CNS and CNS pretreated using DW were 1.9 g/L and 3.6 g/L, respectively. The pretreatment of CNS using DW at 70 °C for 2 h did not remarkably affect the chemical composition but increased the released glucose concentration by 1.9–fold. It is estimated that thermal water softens up the rigid structure of CNS. Various studies have reported that hot water pretreatment at high pressure and high temperature can enhance enzymatic hydrolysis of biomass without the addition of other chemicals [14,28]. The released glucose concentrations were found to be 8.7 g/L, 12.0 g/L and 15.3 g/L at KOH concentration of 1%, 2% and 3%, respectively, and were not significantly affected by KOH concentrations above 3%. In conclusion, KOH concentration suitable for CNS pretreatment was determined to be 3% KOH with high GC and released glucose concentration and low SR.

### 3.2. Optimization of KOH Pretreatment Conditions Using Response Surface Methodology

In order to optimize the pretreatment conditions of CNS, CCD of RSM was carried out. RSM, a statistical and mathematical method, is widely used to minimize the number of experiments and to acquire reliable data [29]. To establish CCD, three factors were divided into five levels as follows: temperature (*X*_1_): 0, 25, 50, 75 and 100 °C; time (*X*_2_): 0, 1, 2, 3 and 4 h and S/L ratio (*X*_3_): 50, 75, 100, 125 and 150 g/L. Zero hours (*X*_2_: –2, Std no.11) means that CNS have not been pretreated. Table 3 shows 20 experiments designed by CCD and their responses. To confirm the repeatability of the experiments, the same experiments were performed 6 times at the center point (Std no. 15–20) [30]. The responses were determined as GC and ED because KOH pretreatment was performed to increase GC by removing other portions (e.g., lignin) of CNS and improve ED of CNS. The ranges of each response were 45.1–84.8% for GC and 12.7–53.8% for ED, respectively.

The model equation for the responses was estimated based on multiple regression analysis of the experimental data.

*Y_GC_* = 73.67 + 6.37 *X*_1_ + 4.70 *X*_2_ − 0.69 *X*_3_ + 0.82 *X*_1_*X*_2_ − 0.084 *X*_1_*X*_3_ + 0.21 *X*_2_*X*_3_ + 0.19 *X*_1_^2^ − 3.34 *X*_2_^2^ + 0.37 *X*_3_^2^ (5)

*Y_ED_* = 43.36 + 5.15 *X*_1_ + 5.81 *X*_2_ − 0.31 *X*_3_ − 0.40 *X*_1_*X*_2_ + 0.16 *X*_1_*X*_3_ − 0.66 *X*_2_*X*_3_ − 0.53 *X*_1_^2^ − 3.51 *X*_2_^2^ − 0.87 *X*_3_^2^ (6)

where *Y_GC_* is glucan content (%) and *Y_ED_* is enzymatic digestibility (%), respectively. *X*_1_, *X*_2_ and *X*_3_ are the independent factors and signify temperature, time and S/L ratio, respectively.

The ANOVA results for each response surface quadratic model are shown in Table 4 and Table 5. The mean squares were computed by dividing the sum of squares by the degrees of freedom [31]. The accuracy of models was described by the F-value [32]. F-values of each model were found to be 10.99 for GC and 9.42 for ED. A *p*-value lower than 0.05 means that the model or model term is significant [33]. The *p*-values of each model were found to be 0.004 and 0.008, respectively. Based on these results, both models were proved to be significant. In addition, it was verified that the three model terms such as *X*_1_, *X*_2_ and *X*_2_^2^ (*p*-value < 0.05) had a significant effect on both GC and ED. The *p*-values of the lack of fit were found to be 0.0593 for GC and 0.0547 for ED, which was not significant for pure error (*p*-value > 0.05). It was confirmed that the predicted models statistically fit the experimental data for the responses [34]. The coefficient of determination (R^2^) should be more than 0.8, and the coefficient of determination approaching 1 means that the experimental value for the response agrees with the predicted value within the designed experimental range [35]. The difference between R^2^ and adjusted R^2^ should be less than 0.2 and the high adjusted R^2^ (>0.75) means that model is statistically acceptable [36]. R^2^ of each model were 0.9082 and 0.8945, respectively, and it was confirmed that the difference between R^2^ and adjusted R^2^ in each model did not exceed 0.2. The coefficient of variation (CV) explains the variance of the data, and the low CV (<10%) demonstrates the accuracy and reliability of the results [37]. The CV of each model was 5.16% and 9.86%, respectively, and both models were proved to have accuracy and reliability. The adequate precision represents the signal to noise ratio and is desirable greater than 4 [38]. Each model was suitable to explore the designed space, showing that the adequate precisions of each model were 13,939 and 12,306, respectively.

**Figure 2:**
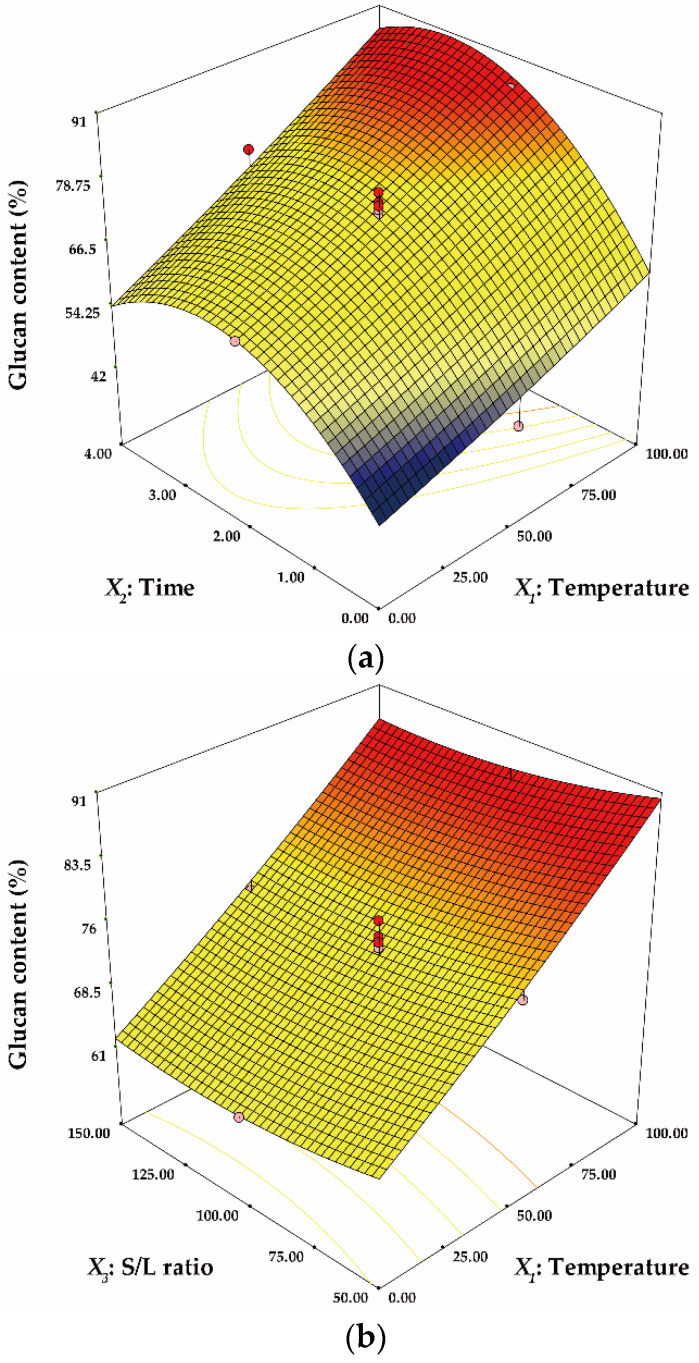

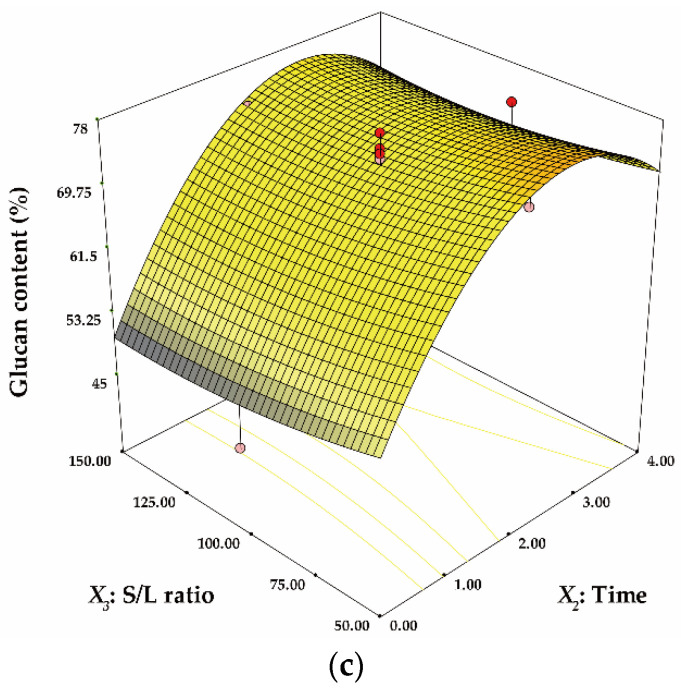
The three-dimensional response surface plot for the interactive effect of temperature and time (**a**), temperature and S/L ratio (**b**) and time and S/L ratio (**c**) on glucan content (GC).

**Figure 3:**
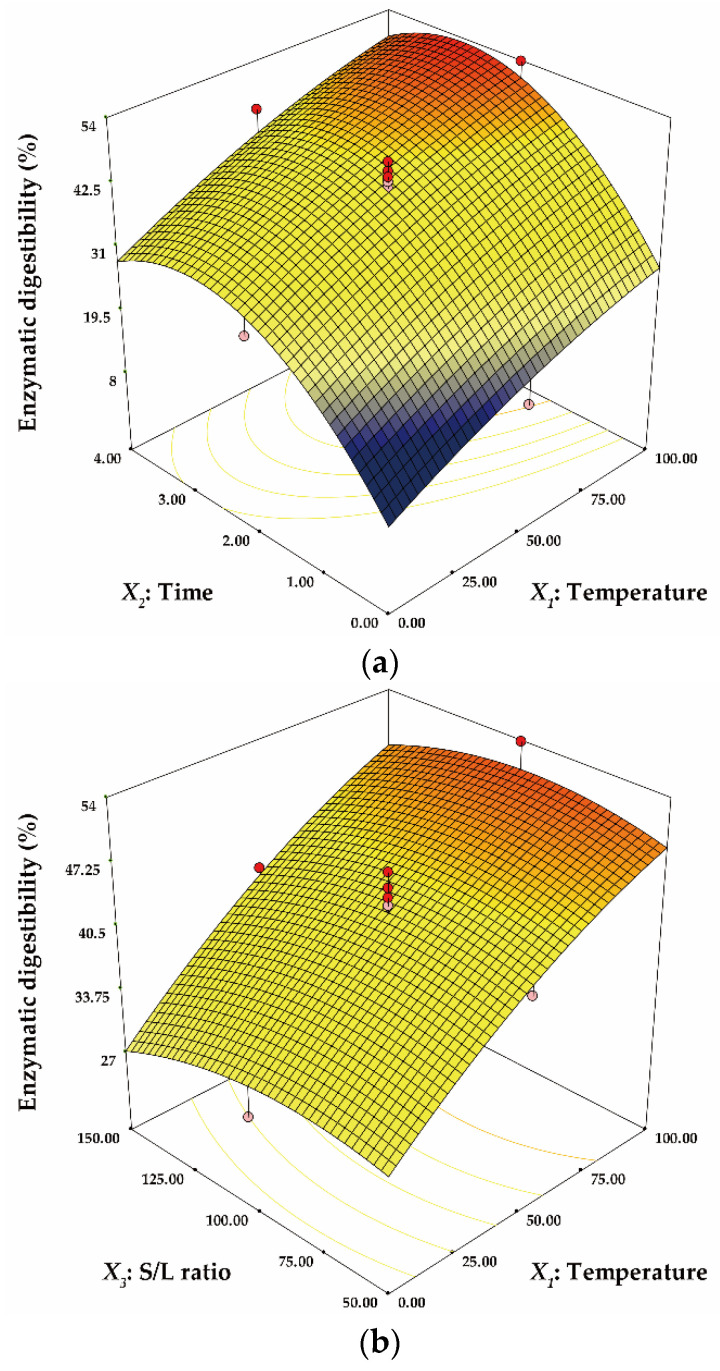

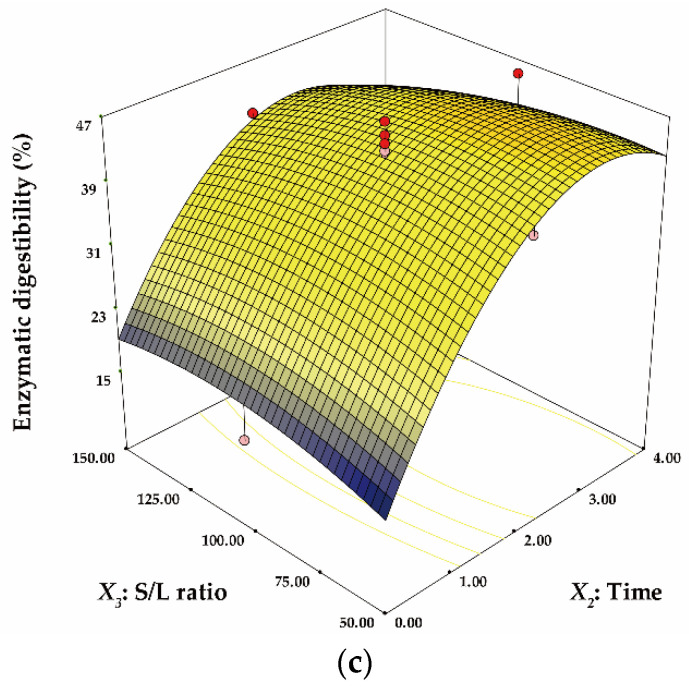
The three-dimensional response surface plot for the interactive effect of temperature and time (**a**), temperature and S/L ratio (**b**) and time and S/L ratio (**c**) on enzymatic digestibility (ED).

Three-dimensional response surfaces were plotted based on the model Equations (5) and (6). The three-dimensional plots are useful for explaining the effect of interactions between independent factors on the response [39]. Figure 2 shows the effect of the interactions between independent factors on GC. In Figure 2a, the minimum GC was estimated as 42.2% at 0 °C for 0 h. GC tended to increase steadily from the minimum point as temperature and time increased. Figure 2b depicts the interactive effect of temperature and S/L ratio on GC. The maximum GC was obtained with 90.3% at 100 °C and 50 g/L. GC was not significantly affected by the S/L ratio and decreased drastically with a lower temperature. Figure 2c portrays that GC was not significantly affected by the S/L ratio and rapidly altered as time changed based on 2.5 h. The effect of the interactions between independent factors on ED is represented in Figure 3. All three-dimensional plots showed similar tendency to those of GC. The effect of temperature and time on ED was observed in Figure 3a. The maximum ED was predicted to be 53.3% at 100 °C for 3 h and the minimum ED was estimated as 3.7% at 0 °C for 0 h. ED increased with increasing temperature and time. In Figure 3b, ED was not significantly affected by the S/L ratio and increased drastically with increasing temperature over the entire S/L ratio range. In addition, in Figure 3c, the S/L ratio did not significantly affect the ED and ED decreased rapidly as time decreased from 3 h.

The purpose of the KOH pretreatment is to improve the enzymatic hydrolysis efficiency [40]. Therefore, numerical optimization was performed by selecting the enzyme digestibility as the most important response (GC: importance level 3 and ED: importance level 5). The results of numerical optimization are shown in Table 6. Optimum reaction conditions for KOH pretreatment of CNS were determined as follows: temperature of 75.0 °C, time of 2.8 h and S/L ratio of 77.1 g/L. Under the optimum conditions, GC and ED were predicted to be 83.3% and 50.0%, respectively. In order to verify the reliability of our predicted model, an actual experiment was carried out under the same conditions as the predicted model. The experimental results show that GC and ED were found to be 83.2% and 48.4%, respectively, proving that our model was suitable to predict the KOH pretreatment of CNS. In conclusion, through the optimization of KOH pretreatment, ED of CNS was determined to be 48.4%, which was improved by 3.8-fold compared to the control group (untreated CNS, ED: 12.7%).

The recent studies that focus on the optimization of alkali pretreatment are summarized in Table 7. The pretreatment of various biomass such as canola straw, *Sida acuta* (Thailand Weed), *Sicyos angulatus*, *Miscanthus*, bamboo, corncob, walnut shell, corn stover, orange peel and spent coffee ground (SCG) were carried out under the determined conditions using alkali reagents (NaOH, NH_3_ and KOH) [17,18,22,41,42,43,44,45,46]. The reported data such as the solid components and enzymatic digestibility before and after the pretreatments were investigated. It was focused on the effect of pretreatment on improved sugar content by enzymatic hydrolysis. Among these, NH_3_ pretreatment have been shown to be effective for high glucose yield after enzymatic hydrolysis. It can easily break the sugar complex in biomass. However, it was not appropriate for a scale-up process due to high capital cost and time [17]. Another pretreatment, such as the NaOH pretreatment, is time-consuming, which could be a bottleneck [17,18,42]. Boonchuay et al. investigated KOH pretreatment and observed that it led to high glucose content from pretreated biomass [43]. It has also been an effective method for high glucose yield after enzymatic hydrolysis. Other reports also confirmed this phenomenon, and it was almost founded in the reaction conditions within 3 h of 3% KOH, 75–121 °C [44,45,46]. The CNS used in this study is rich in carbohydrates that can serve as substrates in the fermentation process. The pretreated CNS recovered about 3.8–fold improved glucose after enzymatic hydrolysis. Unlike other pretreatments, this is advantageous because of its high yield and adequate time. These results show that food wastes such as CNS have the potential to replace sugar cane and corn starch, which are currently utilized in biorefining industries but have food ethics issues. Thus, this study provides evidence for the potential for alkaline pretreatment of CNS for biorefinery systems. This can provide useful information regarding the development of economic and efficient processes using pretreatment systems.

## 4. Conclusions

In this study, the optimum conditions of KOH pretreatment were investigated to improve enzymatic digestibility of CNS using RSM. Statistical models were established to explain the effects of independent factors such as temperature, time, and S/L ratio on responses such as GC and ED. Temperature and time were found to have a relatively more significant effect on GC and ED than S/L ratio. As a result of numerical optimization, the optimum conditions were found as follows: temperature of 75.0 °C, time of 2.8 h and S/L ratio of 77.1 g/L. GC and ED of untreated CNS (control group) were found to be 45.1% and 12.7%, respectively. GC and ED of CNS pretreated under the optimum conditions (experimental group) were found to be 83.2% and 48.4%, respectively. By KOH pretreatment, GC and ED of CNS increased 1.8–fold and 3.8–fold, respectively, our KOH pretreatment had a significant effect on the improvement of enzymatic hydrolysis. Although the ED of CNS was significantly improved, but it was still less than 50%. The drastic improvement of the ED of CNS and the conversion of recovered glucose into more valuable substances through microbial fermentation will be our major study in the near future. The results of this study are expected to provide useful directions for reducing environmental pollution and promoting public health by using food waste as a raw material for biorefinery.

## Figures and Tables

**Table 1:** Factors and their levels for the central composite design (CCD).

Factors	Unit	Symbol	Coded Factor Levels				
			−2	−1	0	1	2
Temperature	°C	*X*_1_	0	25	50	75	100
Time	h	*X*_2_	0	1	2	3	4
S/L ratio	g/L	*X*_3_	50	75	100	125	150

**Table 2:** Chemical compositions of chestnut shells (CNSs).

Main Components	Composition (%, *w*/*w*)
Carbohydrate	
Glucan	45.1–57.8
XMGA ^1^	2.4–5.9
Protein	9.4
Lipid	1.2
Ash	2.4
Others	23.3

^1^ XMGA: the summation of xylan, mannan, galactan and arabinan content in CNS.

**Table 3:** Experimental designs and their experimental data for five-level, three-factor response surface analysis.

Std	Coded Factor Levels			Response	
	*X*_1_	*X*_2_	*X*_3_	GC ^1^ (%)	ED ^2^ (%)
1	−1	−1	−1	64.7	31.0
2	1	−1	−1	76.5	40.3
3	−1	1	−1	66.9	41.2
4	1	1	−1	83.2	46.7
5	−1	−1	1	62.5	31.1
6	1	−1	1	75.2	38.8
7	−1	1	1	66.9	36.5
8	1	1	1	81.5	44.7
9	−2	0	0	61.5	28.0
10	2	0	0	84.8	53.8
11	0	−2	0	45.1	12.7
12	0	2	0	72.9	45.3
13	0	0	−2	75.3	38.8
14	0	0	2	72.4	40.3
15	0	0	0	74.4	44.8
16	0	0	0	71.7	46.4
17	0	0	0	73.1	39.9
18	0	0	0	70.0	41.8
19	0	0	0	73.8	42.9
20	0	0	0	76.4	43.7

^1^ GC: glucan content. ^2^ ED: enzymatic digestibility.

**Table 4:** ANOVA for response surface model of glucan content (GC).

Source	Sum of Square	Degree of Freedom	Mean Square	F-Value	*p*-Value	Remarks
Model	1341.85	9	149.09	10.99	0.0004	significant
*X*_1_	649.43	1	649.43	47.86	<0.0001	significant
*X*_2_	353.97	1	353.97	26.09	0.0005	significant
*X*_3_	7.57	1	7.57	0.56	0.4723	
*X*_1_ *X*_2_	5.35	1	5.35	0.39	0.5443	
*X*_1_ *X*_3_	0.056	1	0.056	0.004151	0.9499	
*X*_2_ *X*_3_	0.36	1	0.36	0.027	0.8734	
*X*_1_^2^	0.87	1	0.87	0.064	0.8049	
*X*_2_^2^	280.53	1	280.53	20.67	0.0011	significant
*X*_3_^2^	3.36	1	3.36	0.25	0.6294	
Residual	135.69	10	13.57			
Lack of fit	111.54	5	22.31	4.62	0.0593	
Pure error	24.15	5	4.83			
Total	1477.55	19				

Coefficient of determination (R^2^): 0.9082. Adjusted R^2^: 0.8255. Coefficient of variation (CV): 5.16%. Adequate precision: 13.919.

**Table 5:** ANOVA for response surface model of enzymatic digestibility (ED).

Source	Sum of Square	Degree of Freedom	Mean Square	F-Value	*p*-Value	Remarks
Model	1282.20	9	142.47	9.42	0.0008	significant
*X*_1_	423.94	1	423.94	28.04	0.0004	significant
*X*_2_	540.22	1	540.22	35.73	0.0001	significant
*X*_3_	1.54	1	1.54	0.10	0.7559	
*X*_1_ *X*_2_	1.31	1	1.31	0.086	0.7749	
*X*_1_ *X*_3_	0.20	1	0.20	0.013	0.9117	
*X*_2_ *X*_3_	3.49	1	3.49	0.23	0.6411	
*X*_1_^2^	7.01	1	7.01	0.46	0.5114	
*X*_2_^2^	309.48	1	309.48	20.47	0.0011	significant
*X*_3_^2^	19.12	1	19.12	1.26	0.2871	
Residual	151.21	10	15.12			
Lack of fit	125.23	5	25.05	4.82	0.0547	
Pure error	25.98	5	5.20			
Total	1433.41	19				

Coefficient of determination (R^2^): 0.8945. Adjusted R^2^: 0.7996. Coefficient of variation (CV): 9.86%. Adequate precision: 12.306.

**Table 6:** Numerical optimization and experimental data for the chestnut shells (CNSs) pretreatment.

**Factors**	**Coded Levels**	**Actual Levels**
Temperature	1.0	75.0 °C
Time	0.8	2.8 h
S/L ratio	−0.8	77.1 g/L
**Response**	**Predicted**	**Experimental**
GC (%)	83.3	83.2
ED (%)	50.0	48.4

**Table 7:** Summary of changes in the chemical properties of various biomass by alkali pretreatments.

Biomass	Pretreatment Conditions	Glucan (%)		XMGA (%)		ED (%)		References
		Bef.	Aft.	Bef.	Aft.	Bef.	Aft.	
Canola Straw	19.3% NH_3_ at 70 °C for 16 h	34.3	43.8	21.7	13.6	19.5	82.4	[17]
	9.2% NaOH at 70 °C for 16 h	34.3	58.5	21.7	11.3	19.5	90.3	
*Sida acuta*	3% NaOH at 60 °C for 9 h	42.7	61.4	16.1	23.3	5.7	22.6	[18]
*Sicyos angluatus*	2% NaOH at 121°C for 10 min	16.4	46.7	6.2	11.3	22.1	55.3	[22]
*Miscnthus*	15% NH_3_ at 60 °C for 24 h	41.3	55.4	28.7	34.6	33.1	71.3	[41]
Bamboo	2% NaOH at 25 °C for 48 h	37	33	16.6	13.2	24.5	47.1	[42]
Corncob	5% KOH at 90 °C for 1 h	42.6	64.5	38.8	31.3	-	82.4	[43]
Walnut shell	3% KOH at 121 °C for 1 h	39.6	35.6	18.5	36.9	6	56	[44]
Corn stover	3% KOH at 121 °C for 1 h	30.6	47.1	26.6	32.5	48	100	[44]
Orange peel	3% KOH at 121 °C for 30 min	25	32.8	17	-	36	87.8	[45]
Spent coffee ground	3% KOH at 75 °C for 2.8 h	9.3	18.9	23.3	47.5	31.6	59.4	[46]
Chestnut shell	3% KOH at 75 °C for 2.8 h	45.1	46.1	5.9	9.5	12.7	48.4	This study

## Data Availability

The data presented in this study are available on request from the corresponding author.
